# Measuring User Similarity Using Electric Circuit Analysis: Application to Collaborative Filtering

**DOI:** 10.1371/journal.pone.0049126

**Published:** 2012-11-07

**Authors:** Joonhyuk Yang, Jinwook Kim, Wonjoon Kim, Young Hwan Kim

**Affiliations:** 1 Graduate School of Culture Technology, Korea Advanced Institute of Science and Technology, Daejeon, Republic of Korea; 2 Department of Electrical Engineering, Pohang University of Science and Technology, Pohang, Republic of Korea; 3 Department of Management Science/Graduate School of Culture Technology, Korea Advanced Institute of Science and Technology, Daejeon, Republic of Korea; Umeå University, Sweden

## Abstract

We propose a new technique of measuring user similarity in collaborative filtering using electric circuit analysis. Electric circuit analysis is used to measure the potential differences between nodes on an electric circuit. In this paper, by applying this method to transaction networks comprising users and items, i.e., user–item matrix, and by using the full information about the relationship structure of users in the perspective of item adoption, we overcome the limitations of one-to-one similarity calculation approach, such as the Pearson correlation, Tanimoto coefficient, and Hamming distance, in collaborative filtering. We found that electric circuit analysis can be successfully incorporated into recommender systems and has the potential to significantly enhance predictability, especially when combined with user-based collaborative filtering. We also propose four types of hybrid algorithms that combine the Pearson correlation method and electric circuit analysis. One of the algorithms exceeds the performance of the traditional collaborative filtering by 37.5% at most. This work opens new opportunities for interdisciplinary research between physics and computer science and the development of new recommendation systems

## Introduction

While various kinds of recommendation methods have been proposed, collaborative filtering (CF) is still the most widely used [1]. Typical CF techniques rely on trust or similarity relationships among users; these are based on either or both *social ties* (i.e., “I trust my friends, and the people that my friends trust”) and *taste similarity* (i.e., “I trust those who agree with me”) [2]. The former uses the social relationship information of users [3], which can be observed on social networking services such as Twitter [4]. The latter constructs implicit network structures for users by observing their shared behavior, such as reading the same books [5], spending time on the same Web pages [6], or purchasing the same items [7]–[10]. Some previous studies have facilitated the convergence of these two approaches through hybrid algorithms that exploit both social relationship and taste similarity [11]–[13].

In line with this, increasing attention is now being paid to network science [14], and the development of network analysis techniques is opening up new opportunities for advances in CF. This is because both social relationships and taste similarities can be easily represented as a graph or network of interconnected users. For example, [13] directly used the social network information of users to distinguish friends and strangers from their neighbors and thus enhance the CF performance. However, the utilization of social network data for CF necessarily requires additional data collection efforts. In addition, the memory-based CF with Pearson correlation (PC), Jaccard or Tanimoto coefficient (TC), or Hamming distance (HD) allows us to calculate only the direct one-to-one similarity between users instead of utilizing the information from both the direct and the indirect relationship structure. This is problematic when the number of items is far greater than the number of users and vice versa. As we observe more zeros in the user–item matrix, the similarity score between users approaches zero–this is called the sparsity problem [15].

Therefore, in this study, we propose a new technique of measuring user similarity: applying electric-circuit analysis (ECA) to the user–item matrix. By doing so, we use the full information about the relationship structure of users in the perspective of item adoption for enhanced CF that overcomes the limitations of the traditional similarity measures such as the PC, TC, or HD. ECA is used to measure the potential differences between nodes or current flow in electrical networks in physics. However, when applied to a circuit-represented consumer graph, ECA can measure the potential differences between pre-adopters and non-adopters for a product. Here, the relative potential values of non-adopters represent the similarity between users - equivalent to the PC, TC, or HD.

Based on a set of experiments, we found that the ECA algorithm exhibits better predictability than traditional user-based CF with other similarity measures. Moreover, we find that ECA and the CF methods with other measures afford different recommendation sets for items. Thus, by combining their results, we could realize a set of hybrid CF models that result in a far better recommendation performance than traditional user-based CF.

Consequently, this paper contributes to the areas of recommender systems in at least two important respects: first, instead of relying on the existing similarity calculation methods, our system employs a model that can incorporate the full relationship structure information among potential recommendation items. Second, our interdisciplinary approach between physics and computer science opens new opportunities for developing new hybrid models with possibly better performance in recommender systems. To the best of our knowledge, this is the first attempt to apply ECA to information retrieval.

## Theoretical Background

### Network Analysis for Collaborative Filtering

CF, the most popular recommendation technique thus far, is the process of recommending relevant items to users on the basis of peer behavior [16]–[18]. In other words, CF is an assortment size-reduction process for supporting agent decisions on the basis of the choices of other agents. Because of its simplicity and powerful predictability, several firms such as Amazon.com, TiVo, Yahoo!, and Netflix have adopted the algorithm in their businesses [8], [19], [20].

Generally, CF implementations are classified into two main groups according to their method of processing data: model-based algorithms and memory-based algorithms [15]. Hybrid CF algorithms that combine memory-based and model-based algorithms constitute yet another class. Model-based algorithms aim to find behavioral patterns among users by using data-mining or machine-learning techniques. Bayesian belief net CF [21], clustering CF models [22], [23], and latent semantic CF models [24] are typical examples. Model-based algorithms usually handle the sparsity problem better than memory-based algorithms and have improved prediction performance. However, they suffer from a trade-off between scalability and predictability [15], involve expensive model-building processes, and are difficult to implement.

In contrast, memory-based algorithms are much easier to implement. The core module of these algorithms is measuring the similarity, or weight, between users. The likelihood of a user adopting or rating a product is estimated using a similarity score calculated from the weighted average of ratings from all other users. The similarity is usually calculated using the PC, TC, or HD. Because of its simplicity and ease of implementation, PC is one of the most widely adopted methods in the CF research community for quantifying the relationship between two different users [15].

One drawback of the method, however, is that the one-to-one correlation calculation cannot incorporate the relationship structure information of users. To solve this problem, there have been attempts to incorporate social network information into CF techniques [3]. Social network information enables us to observe physical interactions among users, so it helps to build better prediction models. Several studies have shown that the use of social network information can enhance the performance of CF techniques [11]–[13]. However, the requirement for additional social network data and the associated computational cost are significant limitations of such an approach.

Alternatively, [9] showed that the CF user–item matrix itself can be represented as a consumer–product bipartite graph, and explained why and when CF works using graph theory [25]: CF predictability increases with the number of *n*-node paths connecting consumers and products sequentially in a consumer–product bipartite graph. Although, such studies are valuable as they expand the boundaries of CF models, their important limitation is that they still largely depend on the PC based similarity measure. In other words, though the user–item matrix is represented as a network, the relationship information within the network is not effectively utilized in their approach. Here, we overcome this limitation using electric circuit theories from physics.

### Electric Circuit Theory

**Figure 1:**
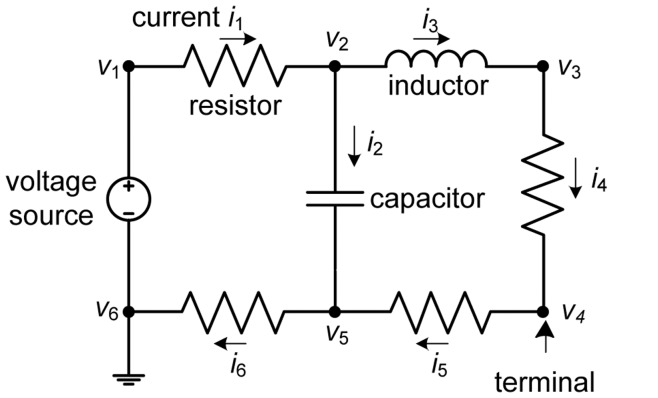
Typical example of an electric circuit.

Because electric circuit theory and analysis methods are relatively unfamiliar topics in the field of computer science, we begin with a brief introduction of ECA before discussing its connection to collaborative filtering. An electric circuit is a closed loop comprising an electrical network, which is an interconnection of electric elements (Figure 1). As a network, an electric circuit has nodes and edges. A node in an electric circuit is defined as a point where at least two electric elements are connected. There is a special node in an electric circuit called a ground node, shown as node *v*_6_ in Figure 1. The ground node is the reference point from which other voltages are measured and is a common return path for the current.

Next, electric elements are regarded as edges. There are two basic types of electric elements in a circuit: active and passive elements. First, active elements, also called sources, generate voltages or currents; that is, active elements generate energy. A voltage source generates a voltage difference between its two terminals, and a current source supplies current that is independent of the voltage across it.

Second, passive elements consume the energy generated by active elements. There are three basic passive elements: resistors, capacitors, and inductors. A resistor is an element with only resistance, i.e., zero capacitance and inductance. The resistance of an electric element, *R*, is the opposition to the passage of a current through the element; the inverse quantity is conductance *G*, i.e. the ease with which a current passes. By Ohm’s law, *R* is defined as the ratio of voltage across an element to the current through it, and the conductance is the inverse; that is, the current-voltage (*I*–*V*) characteristic for resistance can be expressed as 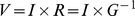. Similarly, the capacitors and inductors also have their own *I-V* characteristics. For simplicity and as the first step, we utilize only the edge property of resistance in the remaining sections of this paper: this restriction could be relaxed in future studies for a more profound investigation.

Active elements generate voltages and currents that produce voltage differences and currents in the other elements. ECA is the process of finding the voltage (*V*) across and the current (*I*) through each element in the network. In other words, ECA finds the electric potential difference between two terminals of an element and the amount of current flowing through that element, which we use to measure the potential differences between pre-adopters and non-adopters for a product in our study.

### ECA for Collaborative Filtering

As mentioned above, ECA identifies all the potential differences between terminals in an electrical network, given that at least one power source is connected to the network. The potential difference between two terminals is directly interpreted as the voltage value of the node. In this study, we use the voltage difference between two nodes as user similarity. That is, the voltage differences between nodes in the circuit can represent a consumer graph, thus replacing the other similarity measures.

A metaphorical example might help in understanding the principle of circuit representation and ECA. Imagine a number of marbles scattered on a flat table. The marbles all look different, and they are interconnected by elastic rubber bands; that is, they form a network. Each rubber band has its own elastic constant, which is inversely proportional to the similarity between the two marbles it connects. The more similar the two marbles are, the less elastic the rubber band is. In other words, the bands hold similar marbles together more tightly. Conversely, the band is more elastic if the marbles are less similar. In the same manner, all the marbles are also connected to the table by rubber bands. This is the same situation as that in the case of the circuit we represent in this paper. For ECA, we pick a marble in the network and lift it up to a certain height. Accordingly, the other marbles are also lifted up, following the picked marble. The height to which a following marble is lifted depends on the elasticity of all the rubber bands going from that marble to (1) the picked marble, (2) the table, and (3) all the other marbles not first picked. The more similar a following marble is to the picked one, the higher the following marble is lifted. ECA measures the exact heights of all the marbles. The following sections formally elucidate this process in detail.

## Materials and Methods

### Circuit Representation

**Figure 2:**
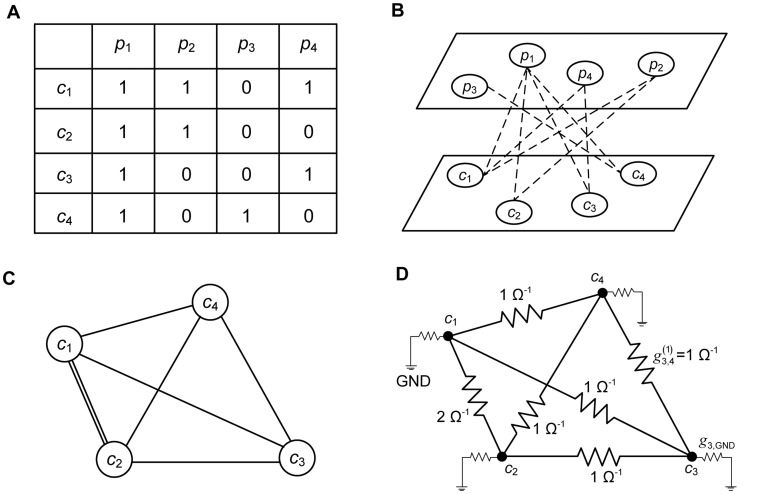
Circuit representation procedure. A: user–item binary matrix; B: consumer–product bipartite graph [9]; C: projected consumer graph [9]; and D: electric circuit representation of the projected consumer graph.

As shown in Figure 2, the user–item binary matrix can be converted into a consumer–product bipartite graph, and then projected onto a consumer graph [9]. The user–item binary matrix in Figure 2(a) shows that *c*_1_ purchased *p*_1_, *p*_2_, and *p*_4_. Similarly, *p*_2_ is purchased by *c*_1_ and *c*_2_. The matrix can be drawn as a consumer–product bipartite graph, as shown in Figure 2(b). We connect a consumer to a product if the consumer purchased the product, so there are no connections between consumers or products themselves. As described in [9], we can now obtain a network of consumers with the relationships among them by projecting the edges of the bipartite graph onto the consumer side. Consumers are connected as shown in Figure 2(c) if there is at least one common purchased product among them. The double line between *c*_1_ and *c*_2_ indicates that they have purchased two products in common. Finally, we convert the consumer graph to an electric circuit, as shown in Figure 2(d).

In the electric circuit, each node represents a consumer or user, the conductance *g_i,j_* between nodes *i* and *j* captures the taste similarity of two consumers, and the conductance *g_i,GND_* between node *i* and the ground captures a characteristic of a consumer such as the annual number of purchases. In this paper, we propose three different ways of defining the conductance, as follows:
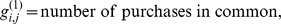


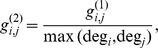


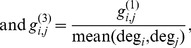
(1)where deg*_i_* is the number of edges outgoing from consumer *i*. As 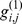 is the number of common purchases between consumers *i* and *j*, it simply quantifies the degree of shared behavior of two consumers in the past. This is under the assumption that a higher frequency of shared behavior between two consumers implies a higher similarity between them. 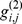 is the normalized shared behavior frequency, obtained by dividing 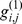 by the maximum degrees of consumers *i* and *j*; it captures the effect whereby more actively purchasing consumers get relatively greater weights with all connected consumers. Similarly, 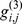 is normalized with the average degree of consumer *i* or *j*.

A characteristic of the consumer, such as the total number of purchases, is modeled as the grounded conductance 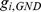 of the corresponding node *i.* In this paper, we propose two ways of defining the grounded conductance, as follows:
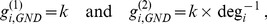
(2)where *k* is a constant. The constant grounded conductance model assumes that all consumers are equally likely to purchase a product. The inverse degree model assumes that consumers with greater numbers of previous purchases are more likely to purchase a product. In this case, a higher grounded conductance indicates a lower voltage level for a node, i.e., a smaller likelihood for purchasing the product.

### Measuring Similarity Using ECA

The first step of any recommendation algorithm is to estimate a likelihood score for each candidate product a user is likely to purchase and to sort the candidates on the basis of that score. This is usually called the top-*k* nearest neighbor (kNN) method. The essential role of ECA is to determine the score as a voltage level. There are two different ways of using ECA for likelihood estimation, according to which type of graph we are dealing with, i.e., a projected consumer graph or a projected product graph. We predict which consumers will purchase a specific product by applying ECA to the projected consumer graph, whereas we predict which product a consumer will purchase with the projected product graph.

Before we explain the method for applying ECA to a projected consumer graph, we need to understand two natural laws in ECA: Kirchhoff’s current law (KCL) and Kirchhoff’s voltage law (KVL). KCL deals with the conservation of electric charge, which implies that the sum of currents flowing into a node is equal to the sum of currents flowing out of that node. In Figure 1, at node *v*_2_, the incoming current *i*_1_ is equal to the sum of outgoing currents, *i*_2_+*i*_3_. KVL deals with the conservation of energy in an electric circuit, which implies that the directed sum of electric potential differences (voltages) around any closed circuit is zero. In Figure 1, for loop *v*_1_−*v*_2_−*v*_5_−*v*_6_, the sum of voltage differences between adjacent nodes, (*v*_1_−*v*_6_)+(*v*_2_−*v*_1_)+(*v*_5_−*v*_2_)+(*v*_6_−*v*_5_), is equal to zero. By applying KCL and KVL to an electric circuit, we can obtain a set of equations describing all branch currents and node voltages. By solving the simultaneous equations, we can find all the node voltages and branch currents. Several methods have been developed for generating these circuit equations systematically, and modified nodal analysis [26] is commonly used.

Therefore, we can apply ECA to a projected consumer graph through the following steps. Note that the opposite case, applying ECA to a projected product graph, is just the dual, so we do not include it in this paper.

1. Represent a user–item binary matrix as a projected consumer graph.
2. Represent the projected consumer graph as an electric circuit.
3. Select a target product and connect unit voltage sources to all the consumers who have purchased the product at that time.
4. Formulate simultaneous equations for the voltage levels of other consumers and the current flow through resistors using KCL and KVL.
5. Solve the equations and obtain all the voltage levels of the consumers. The values represent the likelihood of a consumer purchasing the product: the greater is the voltage value, the higher is the likelihood.

To understand the ECA algorithm more clearly, we applied ECA to the circuit representation of the consumer graph in Figure 2 (see Table 1). With the network, let us suppose we observed that *c*_1_ purchased a newly released *p*_5_. Then we connect *c*_1_ to a power source with a unit voltage, and current flows from it to all the connected consumers. The ECA measures the potential differences of all the other consumers compared with the value of 1 V for *c*_1_. Under the configuration of 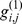 conductance and constant grounded conductance, the voltage at *c*_2_ is 0.615 V, and those at *c*_3_ and *c*_4_ are 0.538 V. This implies that *c*_2_ is more likely to purchase *p*_5_ than *c*_3_ and *c*_4_. This result is intuitive because *c*_2_ is more similar to *c*_1_ than to any other consumer in terms of the historical shared behavior. In the same manner, if we observed that *c*_2_ purchased *p*_5_, then the likelihoods of purchase would be 0.615 V for *c*_1_ and 0.538 V for the other consumers under the same conditions.

**Table 1:** ECA results for the circuit representation of consumer graph in Figure 2(d).

	*c*_1_	*c*_2_	*c*_3_	*c*_4_
*v*_1_	1.000 V	0.615 V	0.500 V	0.500 V
*v*_2_	0.615 V	1.000 V	0.500 V	0.500 V
*v*_3_	0.538 V	0.538 V	1.000 V	0.500 V
*v*_4_	0.538 V	0.538 V	0.500 V	1.000 V

Under the assumption of edge weight 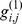 and ground weight 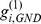.

### Data

We use two datasets: the Movielens dataset, available at http://www.grouplens.org/node/73, and a set of publically inaccessible book transaction logs. The 1 M MovieLens dataset contains 1,000,209 anonymous ratings of 3,952 movies by 6,040 users. The ratings are made on a five-star scale, which is inappropriate for our proposed method that is only applicable to binary data analysis at the moment. Thus, we convert all the ratings to one and null values to zero, as suggested by [27].

Second, individual-level book transaction logs were obtained from an anonymous bookstore chain. The bookstore has both nation-wide offline retailing stores and an online e-commerce Web site. The data we used cover both online and offline sales for three book categories–novels, poetry, and essays–from the first day of 2006 to the last day of 2008. This includes 9,934,309 transaction logs of 1,839,674 distinct registered members, who purchased 62,109 distinct books. Thus, we have a 1,839,674 × 62,109 user–item binary matrix. Because of computational limitations, we used a network-sampling technique, the Metropolis–Hastings random walk (MHRW) algorithm [28]. Unlike random sampling, the MHRW algorithm allows us to sample a certain number of observations without violating the degree distribution of the population. Using the algorithm, we picked 10,000 consumers who purchased more than ten books for the training set.

### Experimental Design

**Figure 3:**
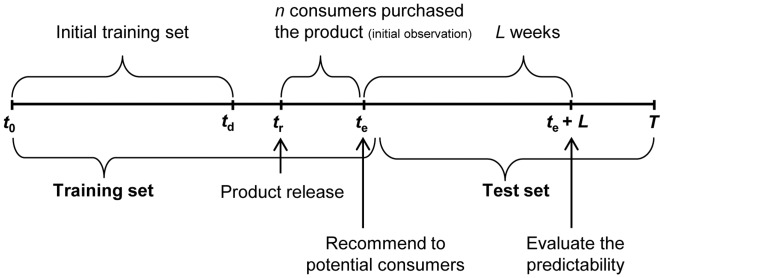
Experimental design.

To evaluate the predictability of the recommendation, we divided the dataset into two groups, namely, the training and test sets. For the MovieLens dataset, we used a randomly selected 80% of the books for the training set and the remaining 20% for the test set. The consumer graph of the training set consists of 6,040 nodes and 17,141,741 edges, which implies a density of 0.940. The mean degree of the graph is 5,700 with a standard deviation of 3,300. For the book transaction logs, we used the first two years, i.e., 2006 and 2007, for the training set and the last year, 2008, for the test set. The basic concept of our experiment is that we recommend to *N* potential consumers that they purchase a product in the test set and evaluate the recommendation results by tracking whether the consumers purchased the product. For example, Figure 3 depicts the recommendation and evaluation procedure for the book transaction logs. First, a new product is released at *t_r_* and we wait until *t_e_* when *n* consumers purchase it, so that we can determine which nodes in the circuit-represented the consumer graph with bias unit voltage. Second, we draw a circuit using the user–item binary matrix at time *t_e_* and connect a unit-voltage power source to the *n* consumers who pre-purchased it. Third, by applying ECA, we obtain the voltage values for all the consumers in the circuit, and sort them in descending order. Fourth, we pick the top *N* consumers and make a recommendation. Fifth, we evaluate the quality of the recommendation *L* weeks later, i.e., at *t_e_*+*L*, by determining whether the recommended consumers purchased the product during *t_e_* or *t_e_*+*L*.

We used three decision-support metrics to evaluate the prediction accuracy [18], [29]: precision, recall, and *F*-measure. Precision is the ratio of the number of hits *h* to the number of recommendations *m*. Recall is the ratio of *h* to the number of actual sales *S* in the test set. *F*-measure is a combination of these two metrics.
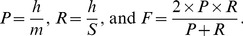
(3)


We performed the experiment for the top-ten best-selling books released between January 2008 and March 2008 for ten different sets of consumer samples. Thus, we obtained 100 precision, recall, and *F*-measures, and averaged them for each recommendation algorithm. A typical consumer graph of the 10,000 nodes has 14,115,000 edges, i.e. density of 0.282, and 2,400 mean degree with a standard deviation of 1,700.

## Results and Discussion

**Figure 4:**
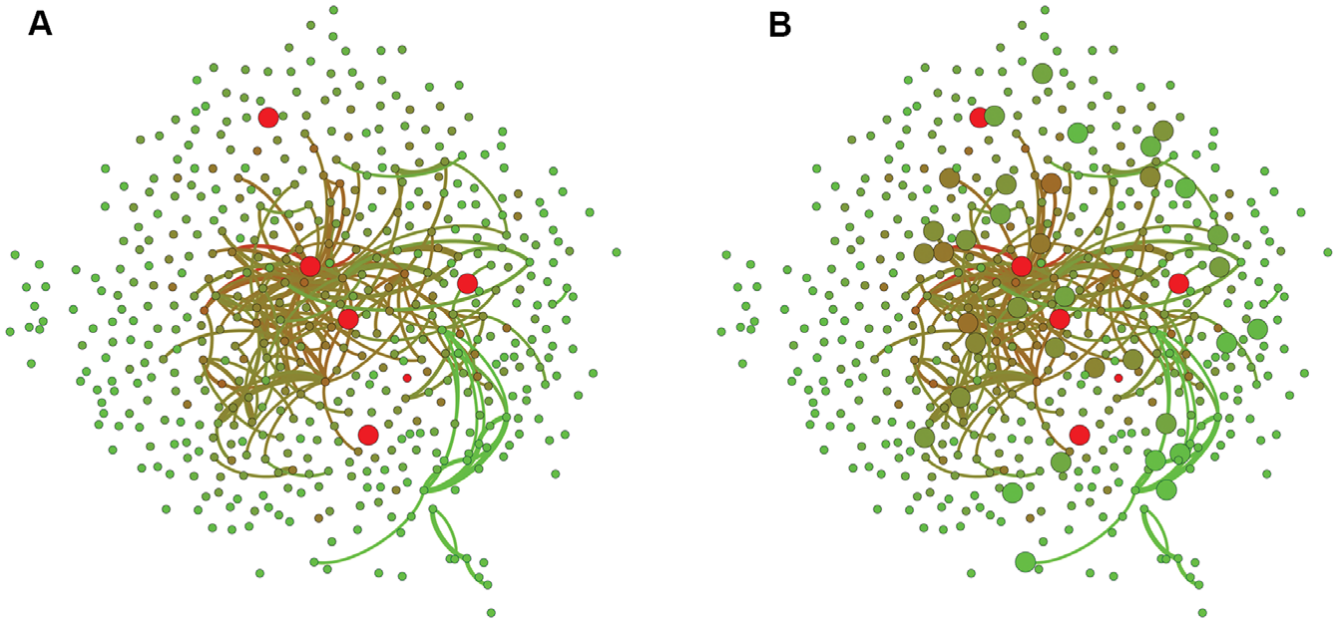
Result of ECA on consumer graph. Nodes for consumers and edges for the number of shared purchases in the past between consumers. For graphical clarity, we dropped the edges with weight less than or equal to five. We used [35] for the graphic tool.

We begin this section by graphically showing the results of ECA on a consumer graph. Figure 4A shows a set of 500 nodes, where each node represents a consumer and five of them purchased a newly released product (in larger circles). The edge weight between two consumers is determined by the number of common products purchased by the two consumers in the past. Next, the node colors represent the potential differences between nodes. The red in the five lager nodes indicates that those nodes are connected to a power source. If the color of a node is closer to red, it has a higher voltage. Conversely, a hue closer to yellow means the node has a lower voltage. Then, Figure 4B shows the purchase status of the same consumers several weeks later. Totally, 41 nodes are in the larger circle, which means that there were 36 additional consumers who purchased the product. Note that the larger nodes are closer to red than the smaller nodes. The remainder of this section formally quantifies and discusses the recommendation quality of the proposed approach.

### Recommendation Quality

In order to compare the recommendation quality of ECA with that of existing methods, we used user-based CF [18], [30] with three different similarity measures: PC, TC, and HD. The benchmark model calculates the score *S_i_* of a potential adopter *i* at the time point *t_e_* as: 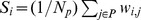, where *P* is a set of pre-adopters, *N_P_* is the number of pre-adopters, and *w_ij_* is the similarity between user *i* and *j*. Then we recommend the product to the top-*N* potential adopters by sorting *S_i_* in descending order. We omit the well-known equations for the three measures to calculate the similarity (see, e.g., [1], [19], [31], [32]). Even though there are some recently developed variations of the basic form of the similarity measure, especially in PC, we could not apply such advancements into our benchmark models. For example, case amplification [18] does not have an effect on the quality metrics in this study because it does not change the order of similarity scores. It rather helps to improve other metrics such as the mean absolute error. The scale and transformation invariant PC [33] is another example we failed to incorporate to our benchmark model owing to its narrow applicability to only voting or rating data. However, we can still find relatively recent papers describing newly developed algorithms that could be compared with the traditional benchmark models used in this study [13], [34]. The choice of a memory-based algorithm as benchmark is attributed to this study’s primary focus on the technique of similarity measure in memory-based CF, and not model-based algorithms without a similarity calculation. One might argue that relatively recent variations of the benchmark model, which generally exhibit better performance, should be used. The main purpose of this study, however, is to provide a technique for measuring similarity or proximity among users, which is the core module for a memory-based CF.

We compare the performance of the benchmark models and six ECA variations for three different inter-consumer conductance models and two different grounded models. For example, ECA1 is conducted under a configuration with 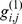 conductance assumptions between consumers and constant grounded conductance 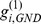.

**Figure 5:**
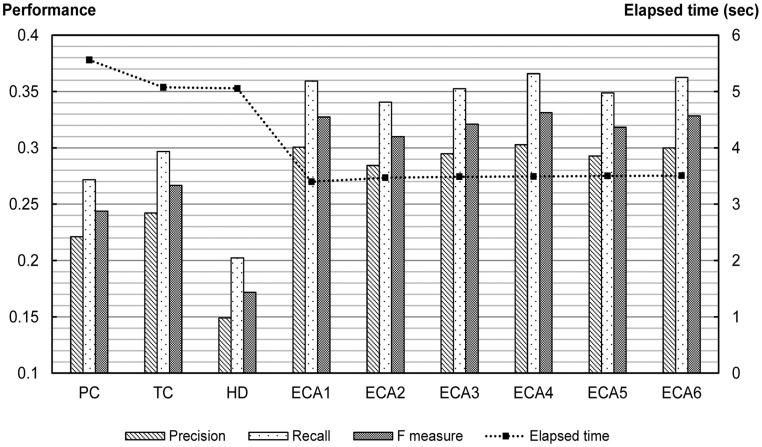
Experimental results for MovieLens dataset. Number of recommendation is 100. There was no significant change in the performance rank among measures when we varied the number to 10 and 50.

**Figure 6:**
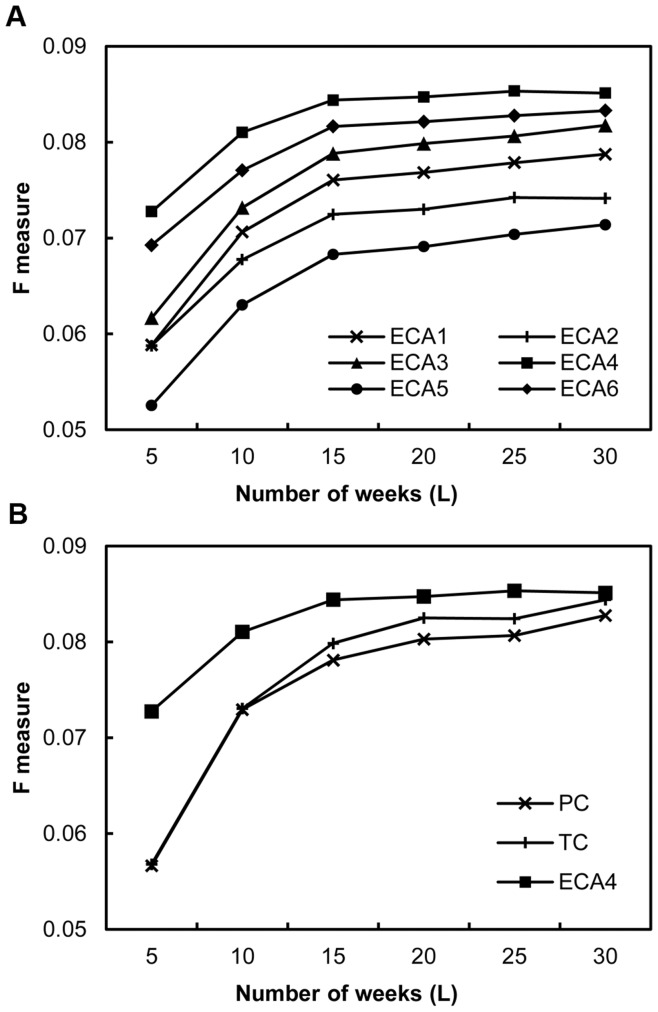
Experimental results for book transaction dataset.

Figure 5 shows the experimental results for the MovieLens dataset. While ECA4, with 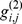
and 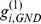, is the highest in all the quality measures, all six ECA variations outperform the benchmark models (i.e. PC, TC, and HD). The relatively smaller average elapsed time needed to make one recommendation is also a strength of ECA. When we varied the number of recommendations to different numbers, such as 10 or 50, to check the robustness of the results, there was no significant change in the performance rank among measures. The results for the book transaction data, shown in Figure 6, confirm what we found in the MovieLens experiment. Figure 6 shows the *F*-measures of all measures listed in an increasing number of weeks required to predict. For example, a value of five for *L* implies that we predict the actual adoption status of consumers five weeks after the similarities among users are measured. This variation in *L* is primarily because the quality metrics, precision and recall, are list-length based. Figure 6A shows that ECA4 is the best among all the six variations as we found in the previous experiment. The gaps between different types of ECA measure do not seem to vary with an increase in *L*. ECA4 is compared with the benchmark models in Figure 6B. For better visibility in the figure, we omitted the HD results, which had far smaller *F*-measure values than the other measures (.009 to.02). While the *F*-measure of ECA4 is higher than that of PC and TC, the gaps between ECA4 and the benchmark models decrease with an increase in *L*. This suggests that ECA performs particularly well when *L* is small, which can be an advantage of ECA from practical point of view. Thus, we can conclude that the ECA measures can be successfully incorporated into recommender systems.

### Graph Density Reduction

Another important quality criterion for recommendation algorithms is scalability [19]. Whereas PC computes only correlations among a node’s *n* neighbors, ECA demands matrix inverse computation at least once. The cost for computing a matrix inverse increases with the cube of the number of nodes, and this could be a drawback of our ECA measures. As shown in Figure 5, applying ECA to time-independent dataset, such as the MovieLens dataset, does not pose a significant scalability problem. The worst case would be where the user-item matrix is updated in real-time whenever there is any additional transaction or adoption. This is problematic because it demands matrix inverse computation each time to make a recommendation. In a practical application, a recommendation system can update its user-item matrix periodically to avoid such computational costs. Moreover, the speed of ECA greatly improves as the density of the consumer graph decreases.

To check the effect of graph density reduction on the ECA prediction accuracy and computation cost, we performed an experiment in which we removed edges whose weights are less than a given threshold *H* and we adjusted the weights of the remaining edges such that 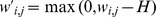. The density reduction threshold was varied from 1 to 14 and we used the book transaction dataset. The other experimental settings were all the same as in the previous section, except we sampled four consumer sets instead of ten. A Linux machine with an Intel Core i7 870 quad-core processor and 8 GB of memory was used.

**Figure 7:**
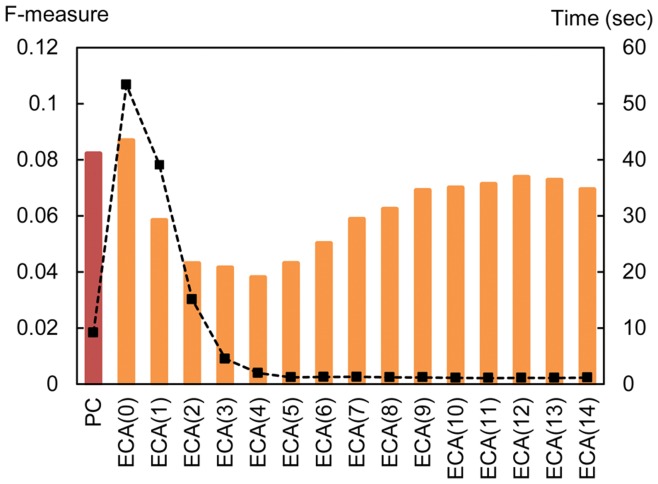
Result of graph density reduction. The red bar represent the Pearson correlation method, and orange bars represent the electric circuit method. ECA(*H*) for graph density reduction with threshold *H*, and ECA(0) represents no graph reduction.

Figure 7 illustrates the results. The bar chart shows the average values of the *F*-measure and the plot represents the average computation elapsed time. The results show that the prediction accuracy of ECA decreases owing to the loss of information until the threshold reaches four, but increases again with *H*. Our explanation for this phenomenon is the effect of noise reduction: by removing meaningless edges, which can be viewed as noise, only meaningful edges remain and the prediction accuracy is improved. Although the *F*-measure did not reach the value of ECA without density reduction, the computation elapsed time is greatly reduced and this could be one of the solutions to the scalability problem of ECA. In-depth analysis of density reduction would be necessary in the future.

### Hybrid Approaches

One additional interesting finding of this study is that the potential consumers are given a recommendation and the hit recommendations differ among the various measures. The smaller intersection areas of the hit sets of different measures imply that there is a greater opportunity for us to improve the performance with a hybrid algorithm that recommends the union hits set. To check the feasibility of such hybrid methods, we compared the number of consumers in the intersection of PC and ECA4 recommendations and the number of hits in common. We used the book transaction dataset.

Table 2 shows the results. Only 11.7 products out of the two top-100 recommendation lists overlapped on an average, and only 3.4 recommendations were hits; that is, the PC and ECA algorithms target different sets of consumers for recommendations, and thus the results show promise for possible complementary use of the two algorithms. For the best case, we can increase the number of hit recommendations for product “1” to 43.6 ( = 27.9+20.2–4.5), i.e., a 56.3% increase compared with CF, if we include all the hits of PC, ECA, and the intersection of the recommendation lists.

**Table 2:** Feasibility check for ECA–PC hybrid algorithms.

Product	A	B	C	D	E
1	27.9	20.2	10.6	4.5	43.6
2	30.2	25.6	14.7	4.9	50.9
3	17.8	14.7	12.5	3.1	29.4
4	9.9	10.7	8.1	1.1	19.5
5	10.3	11.5	8.4	1.7	20.1
6	12.6	12.2	9.1	2.8	22.0
7	8.1	7.7	11.9	1.9	13.9
8	20.3	25.5	13.6	5.2	40.6
9	2.5	2.4	8.2	0.4	4.5
10	17.3	21.1	19.4	7.9	30.5
Mean	15.7	15.2	11.7	3.4	27.5

A: number of items hit out of 100 using the Pearson correlation method; B: number of items hit out of 100 using the ECA method; C: number of the products recommended by both the Pearson correlation and ECA method; D: number of hits out of the common recommendations (C); and E: number of the best possible hits.

**Figure 8:**
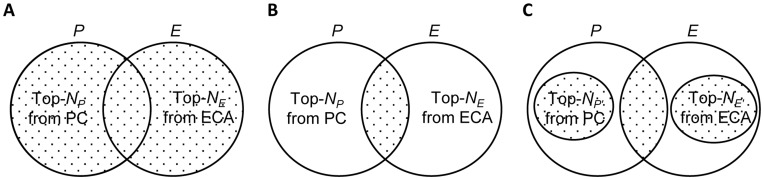
ECA–PC hybrid algorithms. A: hybrid1 (H1) top-*N* split, *n*(A
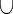
B) = N, where *n*(*P*):*n*(*B*) = *a*:*b* and (*a*,*b*) = {(3,7), (5,5), (7,3)}; B: hybrid2 (H2) top-*N* combination, *n*(*P*')+*n*(*E*')+*n*(*P
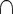
E*) = *N*, where *P*' = *P*−(*P
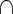
*E), *E*' = *E*−(*P
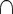
E*), *n*(*P
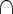
E*):*n*(*P*'):*n*(*P*') = a:b:c, and (a,b,c) = {(1,1,1),(2,1,1), (1,2,1),(1∶1∶2)}; and C: hybrid3 (H3) top-*N* common method, *n*(*P
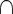
E*) = *N*.

Based on the above findings, we propose four types of ECA–PC hybrid methods heuristically: (1) top-*N* split, (2) top-*N* combination, (3) top-*N* common, and (4) weighted average. Figure 8 depicts the first three approaches graphically. First, the top-*N* split approach (Figure 8A) generates two sorted consumer lists, one from PC and one from ECA, and then picks the top-*N*_A_ consumers from PC and the top-*N*_B_ consumers from ECA, where *N*_P_+*N*_E_ = *N*. We varied the ratio in three ways: the mixing ratio of PC versus ECA for hybrid 1a (H1a) is 3∶7, H1b is 5∶5, and H1c is 7∶3.

Second, the top-*N* combination approach (Figure 8B) is similar to the top-*N* split, except that it allocates a portion to the consumers in the intersection area. The mixing ratio of intersection versus PC versus ECA is 1∶1∶1 for H2a, 2∶1∶1 for H2b, 1∶2∶1 for H2c, and 1∶1∶2 for H2d. Third, the top-*N* common approach (Figure 8C) picks the top-*N* consumers, listed in descending order, from PC and ECA.

Finally, the weighted average approach simply takes the average values for PC and ECA for a consumer with varying weights:
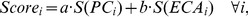
(4)where,



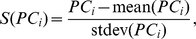


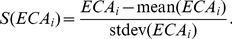
(5)


The PC versus ECA weight ratio (*a*,*b*) is 3∶7 for H4a, 5∶5 for H4b, and 7∶3 for H4c.

Table 3 shows the performance evaluation results for the hybrid algorithms. Most hybrid algorithms exceed the performance of both PC and ECA4. The best performance among the hybrid algorithms is H3, top-*N* common. The precision of H3 is 29.1% higher and the recall is 37.5% higher than that of PC. However, we admit that it is not clear why H3 outperforms the other hybrid measures at the moment. The development of better hybrid algorithm and understanding the underlying mechanism of the different hit sets would constitute challenging and promising future work.

**Table 3:** Performance of ECA–PC hybrid algorithms.

	Precision	Recall	*F-*measure
PC	0.1569	0.0562	0.0828
ECA4	0.1516	0.0592	0.0851
H1a	0.1558	0.0593	0.0799
H1b	0.1571	0.0589	0.0797
H1c	0.1559	0.0571	0.0775
H2a	0.1697	0.064	0.0866
H2b	0.1776	0.0676	0.0911
H2c	0.1642	0.0606	0.0823
H2d	0.1669	0.0639	0.0861
H3	0.2025	0.0773	0.104
H4a	0.1619	0.0585	0.0795
H4b	0.159	0.0572	0.0779
H4c	0.1579	0.0566	0.0771

### Conclusions

The study of recommender systems is an important research area as decision makers are now faced with far more abundant information and an increasing assortment of choices. In this paper, we proposed and analyzed a new, but preliminary, algorithm that can measure the similarity between individuals in product consumption using electric circuit analysis (ECA). Specifically, we represent a consumer graph [9] as an electric circuit, and apply ECA to the circuit representation of the consumer graph. By doing so, we measure the potential differences between pre-adopters and non-adopters for a product and the value allows us to determine recommendation priorities. We also propose four distinct hybrid approaches to enhance the predictability of the algorithm by combining the recommended sets from PC and ECA.

The experimental results show that ECA can be an appropriate and performance-enhancing module for recommender systems. In particular, ECA outperforms all the benchmark models with the MovieLens dataset and one of the proposed ECA–PC hybrid algorithms surpasses the performance of traditional PC by 37.5% in recall. In addition to the development of an advanced recommendation algorithm, this paper has two important implications. First, by using ECA, we can fully employ the network information among consumers or products instead of relying on the existing one-to-one similarity calculation methods such as PC or TC. Second, by applying ECA to management information retrieval, we can open new opportunities for interdisciplinary research among different academic disciplines for resolving the problems in information science.

Several interesting research issues can be addressed in future extensions of this work. First, in addition to conductance, which we actively used in this paper, there are unexplored electric circuit elements such as capacitors and inductors. The electric characteristics of these elements are promising in terms of consumer-behavior modeling. Second, an electric circuit, as well as its analysis, is necessarily dynamic. Dynamic link prediction [10] on consumer–product graphs can be implemented using ECA. Third, one can extend the ECA measure to be applicable to rating or voting data. Fourth, as mentioned in the previous section, understanding the underlying mechanism of the different hit sets among various measures would open great opportunities to develop hybrid algorithms. Finally, although we estimated the proximity of consumers using implicit behavioral relationships in this paper, one can directly incorporate explicit social relationship data into circuit-representation processes. By doing so, a new ECA-based socio-metric index or information diffusion model could be developed.
